# The surging role of Chromogranin A in cardiovascular homeostasis

**DOI:** 10.3389/fchem.2014.00064

**Published:** 2014-08-14

**Authors:** Bruno Tota, Tommaso Angelone, Maria C. Cerra

**Affiliations:** Department of Biology, Ecology and Earth Sciences, University of CalabriaArcavacata di Rende (CS), Italy

**Keywords:** Chromogranin A, Vasostatin 1, Catestatin, Serpinin, adreno-sympathetic control, cardioprotection, vasoactive peptides, endothelial signaling

## Abstract

Together with Chromogranin B and Secretogranins, Chromogranin A (CGA) is stored in secretory (chromaffin) granules of the diffuse neuroendocrine system and released with noradrenalin and adrenalin. Co-stored within the granule together with neuropeptideY, cardiac natriuretic peptide hormones, several prohormones and their proteolytic enzymes, CGA is a multifunctional protein and a major marker of the sympatho-adrenal neuroendocrine activity. Due to its partial processing to several biologically active peptides, CGA appears an important pro-hormone implicated in relevant modulatory actions on endocrine, cardiovascular, metabolic, and immune systems through both direct and indirect sympatho-adrenergic interactions. As a part of this scenario, we here illustrate the emerging role exerted by the full-length CGA and its three derived fragments, i.e., Vasostatin 1, catestatin and serpinin, in the control of circulatory homeostasis with particular emphasis on their cardio-vascular actions under both physiological and physio-pathological conditions. The Vasostatin 1- and catestatin-induced cardiodepressive influences are achieved through anti-beta-adrenergic-NO-cGMP signaling, while serpinin acts like beta1-adrenergic agonist through AD-cAMP-independent NO signaling. On the whole, these actions contribute to widen our knowledge regarding the sympatho-chromaffin control of the cardiovascular system and its highly integrated “whip-brake” networks.

## Introduction

The granins are a structurally and functionally related family of proteins including Chromogranin A (CGA), Chromogranin B (CGB) and Secretogranins (SG) II-VII which are stored in secretory (chromaffin) granules of the diffuse neuroendocrine system and released together with noradrenalin and adrenalin (Winkler and Fischer-Colbrie, 1992; Montero-Hadjadje et al., 2008; Bartolomucci et al., 2011). Detailed information on chromosomal positions, genomic structure, cDNA, and proteins encoded by their three paralogous genes, that likely arose by gene duplication within the vertebrate lineage, has been reported by Mahata et al. (1996), Zhang et al. (2011) and Bartolomucci et al. (2011). The adrenal medulla and the adrenergic terminals are a main source of granins which are therefore been employed as markers of the sympatho-adrenal neuroendocrine (SAN) activity. Granins within the vesicle stabilize the core osmotically by binding catecholamines (CAs) and ATP, and are also co-stored with neuropeptide Y (NPY), the cardiac natriuretic peptide hormones (NPs), several prohormones and their proteolytic enzymes (Videen et al., 1992). Their partial processing to biologically active fragments together with milieu acidification contribute to the maturation of the granule. The mechanisms of granin sorting into regulated secretory pathway granules, including the CGA domains that are required for directing it into the secretory granules, as well as the CGA N- and C-terminus that may have targeting information, have been summarized by Bartolomucci et al. (2011). Among granins, the soluble acidic 439- residue long CGA is the most abundant protein of the granule, accounting for almost 50% of its soluble protein content, and is the most studied member, being considered the major indicator and multifunctional effector of the SAN tone (Cryer et al., 1991; O'Connor et al., 2008). Its wide spectrum of biological and physio-pathological activities ranges from intracellular to organ and system levels.

**Figure 1:**
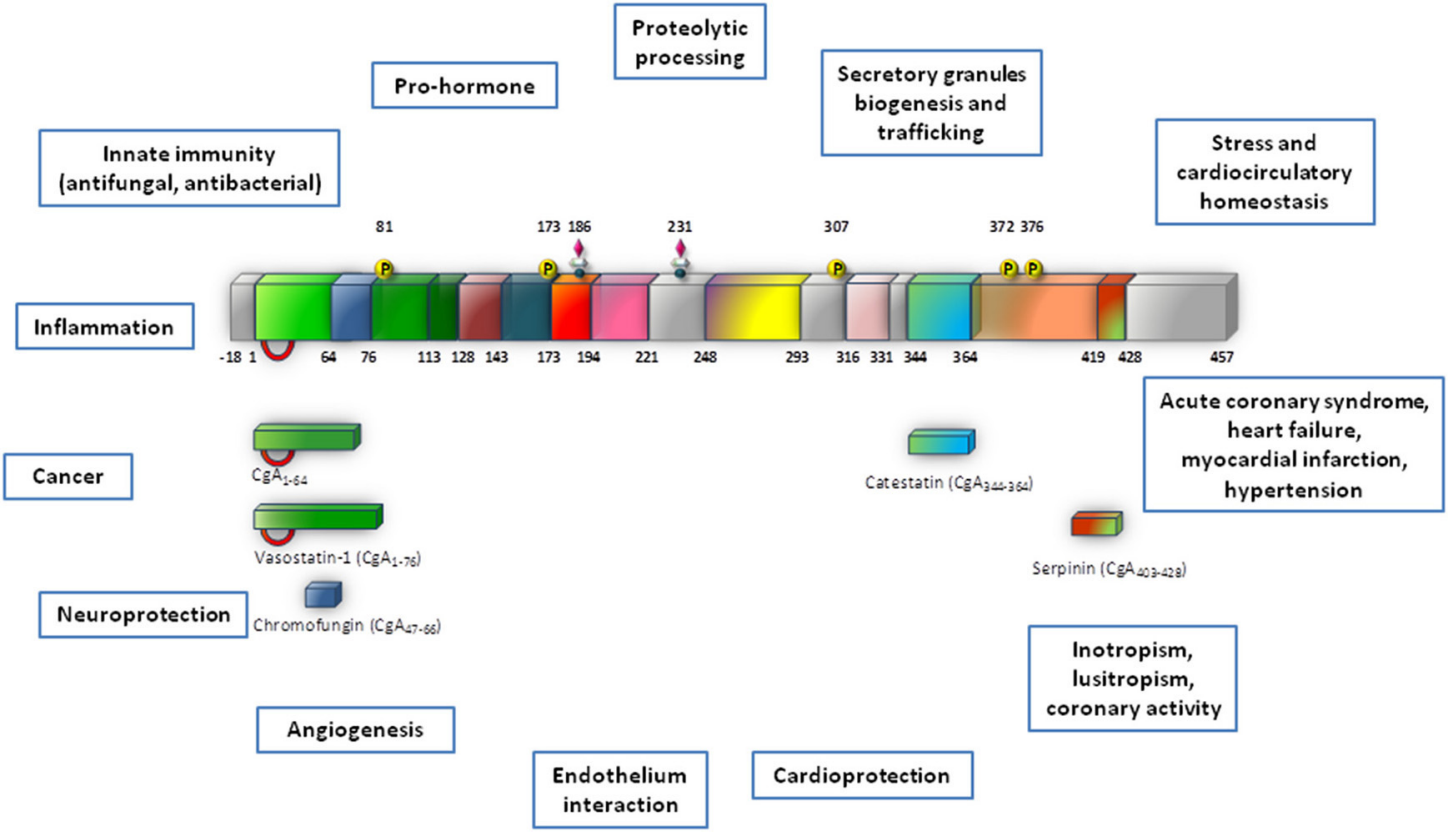
**This synopsis illustrates Chromogranin-A (CgA) processing and biological activities which are reviewed in this volume (modified from Angelone and Tota, 2012)**. This provides a cardiovascular dimension for CgA, with important outcomes in terms of biology, physiology and clinics.

A fundamental intracellular function of CGA is its granulogenic role in the formation of dense core secretory vesicles in (neuro) endocrine cells (Kim et al., 2001; Kim and Loh, 2006). In fact, CGA depletion by gene targeting causes reduction of adrenal chromaffin granules in number, size, and electron density, as well as disruption of transmitter secretion from the regulated pathway (Mahapatra et al., 2005). Together with other granins, CGA binds to the inner layer of the vesicle membrane affecting the release of calcium from secretory granules to the cytosolic exocytotic machinery through the inositol 1,4,5-trisphosphate receptor/Ca^2+^ channel (Yoo et al., 2002). CGA has a long evolutionary history and CGA-like proteins have been detected in mammals, birds, amphibians, fish (including CGA mRNA in zebrafish) and arthropods (Xie et al., 2008). In addition to its expression in the neuroendocrine cells, as detailed below, CGA has also been detected in other cell types, including the myocardiocytes of various vertebrate species, e.g., amphibians (Krylova, 2007), rodents (Steiner et al., 1990; Biswas et al., 2010; Pasqua et al., 2013) and humans, particularly in patients affected by cardiomyopathy and heart failure (HF: Pieroni et al., 2007). In normal conditions, upon stimulation of regulated secretion, CGA is exocytotically released in the extracellular space and then in the circulation, thus being able to exert systemic and/or organ and tissue modulatory effects (Helle et al., 2007; Bartolomucci et al., 2011; Angelone et al., 2012a and references therein). These actions are mainly related to the prohormone/cytokin ability of CGA to undergo a finely regulated clevage. That is, following stimulus- and differential cell-type-specific or tissue-specific proteolytic processing at dibasic sites, CGA generates within secretory vesicles several peptides in aggregate which can then exert relevant modulatory actions on endocrine, cardiovascular, metabolic, and immune systems through both direct and indirect SAN interactions (Metz-Boutigue et al., 1993; Parmer et al., 2000; Taupenot et al., 2002). The CGA biologically active fragments include the amino terminal cardio and vasoactive vasostatin 1 (VS-1), the antimicrobial chromacin, the dysglicemic peptide pancreastatin (PST), the parathormon release modulator parastatin, the catecholamine release inhibitor catestatin (CST), and the recently discovered serpinin (see Figure 1).

With reference to the cardiovascular focus of this review, we will illustrate the actions of both circulating CGA and its three fragments, VS-1, CST and serpinin (Tota et al., 2010, 2012), which exert a broad spectrum of regulatory influences on the cardio-circulatory system. Although nothing is known on how these activities and the underpinning proteolytic events are spatio-temporally coordinated, it is conceivable that both the systemic actions of CGA and those induced by the CGA-derived peptides at organ/tissue (heart and vessels) level may be synergically implicated in circulatory homeostasis, coordinating and counteracting SAN overactivity under normal and perturbed conditions. Namely, these substances can operate as “integral” controller components, bringing the controlled variable back to set “point” at any steady-state disturbance, according to the Koeslag et al.'s (1999) concept of the “zero steady-state error” homeostasis achieved by pairs of counter-regulatory hormones (originally applied for CST and PST). Before discussing this issue, for the non-expert reader we will very briefly summarize the physio-pathological implications of heightened SAN activity.

## Physio-pathological aspects of SAN overactivation

**Figure 2:**
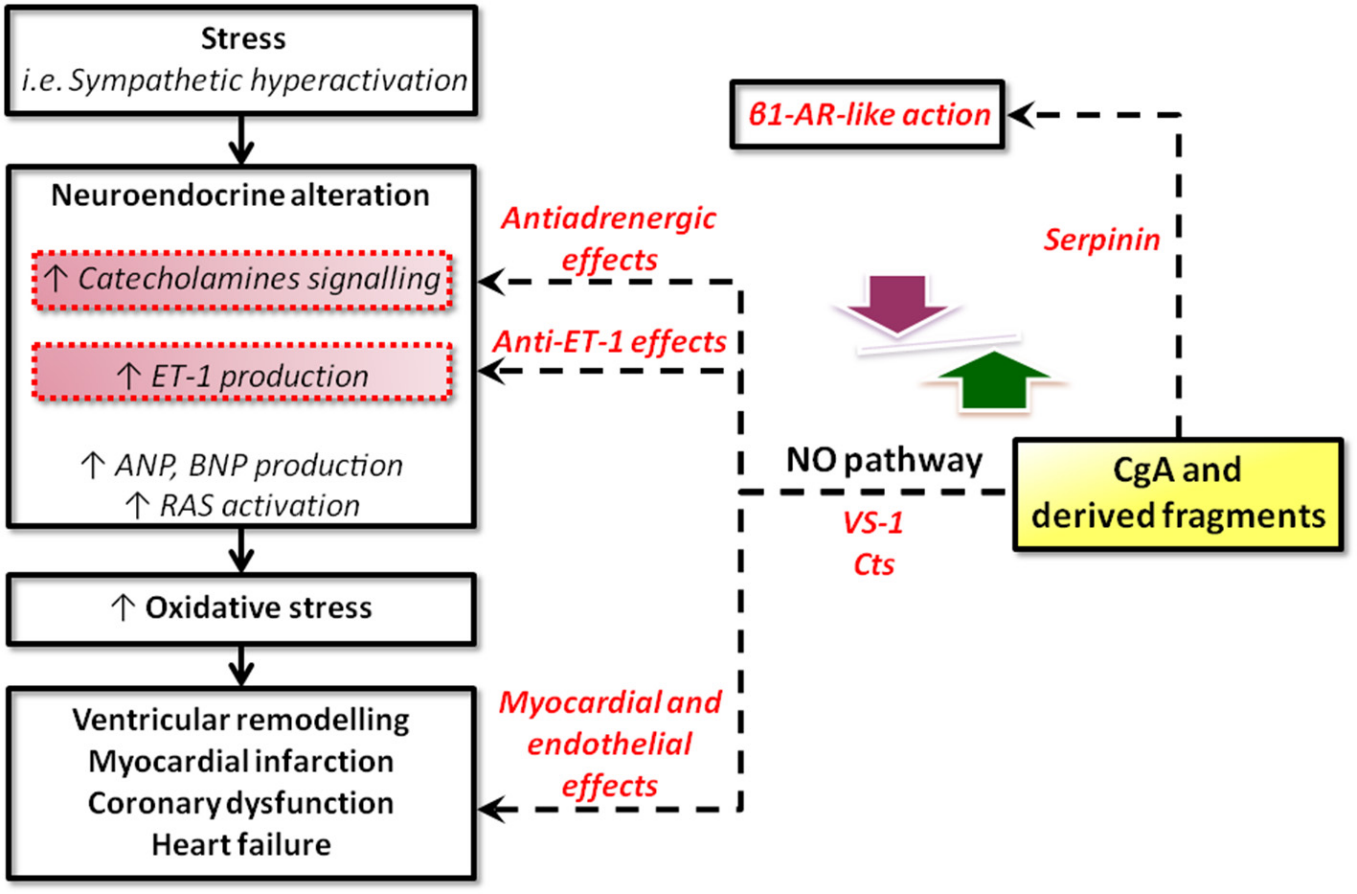
**Schematic representation of the possible sites for intervention of CgA and its derived peptides in heart failure**. CgA and its fragments could operate at two, non-exclusive, levels: systemic and local. At the systemic level, CgA may work together with other factors (catecholamines, ANGII, cytochines, chemochines, etc) in the stress response, as in the case of the neuroendocrine scenario activated in CHF. At the local (heart) level, systemic and/or intracardiac physical and chemical stimuli could trigger CgA processing to generate cardioactive peptides, i.e., VSs, CST, serpinin (modified from Angelone et al., 2012a).

There is a wide spectrum of SAN-induced actions on the circulation, ranging from blood coagulation, platelet adhesiveness, smooth muscle cell hyperplasia, arterial wall tone, hyperlipidemia, denervated myocardium etc. to immunologic responses in relation to cardiovascular changes. Therefore, it is not surprising that SAN overstimulation (the “adrenergic storm”) may act on a number of different targets, directly impinging the heart and the vasculature. The corresponding physio-pathological changes have long been known, ranging from the experimental necrotic damage induced by CAs in the rodent heart (Samuels, 2007) to the extensive clinical evidence showing that the initial heart response to prolonged SAN overactivity leads to compensatory remodeling, cardiac hypertrophy and, if the stress will overwhelm the system, HF (Chien et al., 1991 and references therein). If left uncontrolled, the SAN overactivation may result to be more deleterious than the actual stress placed on the heart. In human HF, chronic heightened adrenergic activation, mainly *via* CAs signaling, has adverse prognostic significance, accelerating the pathological processes (Cohn and Yellin, 1984). Apart from being of clinical relevance, these cardiovascular studies have provided the rationale for anti-adrenergic drug therapy, including the beta-adrenergic-blockers, still amongst the most used drugs. The growing evidence regarding the emerging cardiovascular role of CGA and CGA-derived VS-1, CST and serpinin may represent the next breakthrough in this field (see Figure 2).

## The cardio-circulatory profile of full-length CGA

Circulating CGA (normal values: 0.5–2 nM: Helle et al., 2007; Crippa et al., 2013) increases under conditions of stress-elicited SAN over-activation and physio-pathological conditions, e.g., chronic inflammation, neuroendocrine tumors, acute coronary syndromes and chronic HF. Therefore, CGA plasma levels have been used as prognostic indicators in these conditions (Helle et al., 2007; Angelone et al., 2012a; D'amico et al., 2014). It is important to note that pertinent information on plasma levels of CGA and its derived peptides can be best obtained by serological studies that in addition to processing-independent radioimmunoassays include also region-specific processing-dependent assays. In fact, only the latter can allow to analyse the plasma levels of the various granin-derived fragments which exhibit relevant and sometime opposite biological functions and prognostic significance (Crippa et al., 2013; Goetze et al., 2014). The CGA *in vivo* long half-life (~18 min) and its relatively elevated circulating concentrations (also under normal conditions), reduce the possibility of false measurements and facilitate blood collection, pre-analytic handling and final determinations (O'Connor et al., 1989).

Plasma CGA concentrations are increased up to 10–20 nM (500–1000 ng/ml) in patients with essential hypertension (Takiyyuddin et al., 1995), myocardial infarction (Omland et al., 2003), acute destabilized HF (Dieplinger et al., 2009), acute coronary syndromes (Jansson et al., 2009), chronic HF (Ceconi et al., 2002) and decompensated hypertrophic cardimyopathy (Pieroni et al., 2007). As firstly documented by Ceconi et al. (2002), circulating CGA levels significantly parallel the severity of the dysfunction, representing an independent predictor for mortality. Accordingly, from a clinical point of view, CGA is now emerging as a potentially new diagnostic and prognostic cardiovascular biomarker independent from conventional markers.

Studies in twins indicated that basal plasma CGA concentration is highly heritable (Takiyyuddin et al., 1995). Compared with age-matched normotensive counterparts, patients with essential hypertension show increased plasma CGA and an increased release of stored CGA in response to adrenal medullary stimulation by insulin-evoked hypoglycemia (Takiyyuddin et al., 1995). Among others, these observations confirm the correlation between CGA and SAN activity.

A relevant circulatory function of CGA is related to the regulation of endothelial barrier (Ferrero et al., 2004) and tumor-induced vascular remodeling (Veschini et al., 2011). Both CGA and VS-1 are potent inhibitors of the proangiogenic Vascular Endothelial Growth Factor (VEGF), as well as the thrombin-induced endothelial cell permeability (Ferrero et al., 2004) and inhibit the TNF-elicited changes on endothelial cells, i.e., gap formation, disassembly of vascular endothelial-cadherin adherence junctions and vascular leakage (Ferrero et al., 2004; Dondossola et al., 2011). Systemic administration of CGA (1 μg) to lymphoma-bearing mice potently reduces the TNF-elicited penetration of a synthetic dye (patent blue) in tumor tissues (Dondossola et al., 2011), confirming previous observations that CGA can affect host/tumor interactions (Colombo et al., 2002).

Recently, Crippa et al. (2013) have reported in healthy subjects the presence of biologically relevant plasma levels of full-length CGA, CGA 1–76 (antiangiogenic) and fragments lacking the C terminal region (proangiogenic) and have demonstrated that blood coagulation triggers a thrombin-dependent almost complete conversion of circulating CGA into fragments lacking the C- terminal region. This uncovers a novel role of CGA as an angiogenesis activator which, under conditions of perturbed angiogenesis (wound healing, cancer, etc.), can contribute to circulatory and vascular homeostasis through the opposite angiogenic effects of its fragments (possibly VS-1 and CST) generated by tightly spatio-temporally regulated proteolysis.

A similar modulatory strategy exerted by CGA has been suggested at the heart level on the basis of morphological, biochemical and physio-pharmacological evidences briefly summarized below.

### The intracardiac localization of CGA

Using immunohistochemical technique, Steiner et al. (1990) demonstrated in the myoendocrine granules of the rat heart the co-localization of CGA and ANP. Their immunoblotting finding suggested a more extensive myocardial CGA processing compared to that of the adrenal medulla. As remarked by Miserez et al. (1992), an additional source of cardiac CGA and/or CGA-derived fragments may result from the nerve termini innervating the heart. Weiergräber et al. (2000) showed that CGA was also present in rat Purkinje conduction fibers, in both rat atrium and ventricle, as well as in H9c2 rat cardiomyocytes. More recently, Biswas et al. (2010) confirmed the presence of CGA, as well as that of CGB and SG in the secretory granules of the mouse myocardium.

Importantly from a physio-pathological point of view, Pieroni et al. (2007) provided immunohistochemical evidence of CGA-positive intracellular staining in the human myocardium. They used confocal microscopy to show that in ventricular cardiomyocytes of dilated and hypertrophic human hearts CGA is colocalized with Brain Natriuretic Peptide (BNP). RT-PCR corroborated this finding documenting the myocardial presence of CGA-mRNA, while ELISA assays with four different monoclonal antibodies allowed to measure more than 0.5 μg of CGA per gram of left ventricular myocardial tissue. Assuming a constant myocardial release of CGA and considering that the plasma half-life of CGA is 18.4 min Corti et al. (1996), Pieroni et al. (2007) postulated a significant cardiac contribution to the increased circulating CGA levels reported in their patients. The possible correlation of CGA with the NPs system could contribute to regulate cardiovascular function through short- and long-term depressing influences, inducing tonic vasodilation, hypotension and cardioprotection against adreno-sympathetic hyperactivation. Moreover, because of the strong association between plasma CGA/NPs concentrations and the degree of hemodynamic dysfunction in HF, both hormones have been used as promising prognostic indicators of the severity of HF (see Dieplinger et al., 2009 and references therein). Furthermore, the significant correlation between CGA levels and left ventricle end diastolic pressure may implicate for myocardial CGA the operation of stretch-elicited release and transcriptional up-regulation mechanisms similar to that reported for BNP (Tota et al., 2010 and references therein).

In the rat heart, intracardiac presence and processing of CGA were biochemically demonstrated by Glattard et al. (2006). By submitting the RPHPLC purified CGA-immunoreactive fractions from cardiac extracts to western blot and MS analysis (TOF/TOF technique), the authors characterized four endogenous N terminal CgA-derived peptides, i.e., CGA4–113, CGA1–124, CGA1–135 and CGA1–199, containing the VSs sequence. The intact CGA was also detected among these and other C-terminal truncated fragments. It is important to note the cell-specific feature of this proteolytic CGA fragmentation, as contrasted by the rat adrenal gland in which almost no intact CGA is found; on the other hand, pancreatic beta cells produce betagranin corresponding s to the N-terminal portion of CGA (Hutton et al., 1987). The comparison of these and other observations seems to indicate that in the heart the maturation process can be incomplete and specific. It is of particular interest the finding, more recently confirmed by Pasqua et al. (2013), that the cardioactive motif (the VS-1 sequence or a portion of it) is present among the low-molecular-mass fragments identified. Therefore, the possibility exists that, under normal or stressfull conditions the heart responds to a specific physical (e.g., stretch) or chemical (e.g., CAs) stimulus activating proteolytic CGA processing with subsequent increase in lower-molecular-mass cardioactive fragments. This supports our working hypothesis (Pasqua et al., 2013, and references therein) that CGA, in addition to its endocrine and systemic role, can exert a direct autocrine/paracrine modulation on the heart.

### Physio-pharmacological evidence of direct CGA cardioactivity

Using isolated and Langendorff perfused hearts of normotensive and spontaneously hypertensive rats (SHR), Pasqua et al. (2013) have shown for the first time that the exogenous full-length CGA (1–4 nM) directly affects myocardial contractility and coronary vasomotion (dilation) by Akt/NOS/NO/cGMP/PKG pathway. At the same time, the rat heart in response to hemodynamic and chemical (β-adrenergic and ET-1) excitatory stimuli generates CGA fragments, including the cardioactive VS-1. Therefore, this evidence, which conceptually interlocks the systemic (endocrine) and intracardiac (paracrine/autocrine) actions of full-length CGA and its derived cardioactive peptides, provides a rationale for future investigations on the putative multilevel switches of the SAN/CGA axis that may operate under normal and physio-pathological conditions. In this perspective, here below we will briefly illustrate the cardiovascular profiles of VS-1, CST and serpinin, that have been documented in rodent heart preparations. For space economy, we will not discuss the cardiac effects that VS-1 and CST exert on cold vertebrate hearts (frog and eel) which confirm the peptide profiles observed in the rodent heart (see for references, Tota et al., 2010). Our aim is to highlight the intriguing characteristics of a protein like CGA which has revealed itself to function both as a pleiotropic hormone and a cytokine.

## Cardiac actions of VS-1

Numerous studies conducted by our group have demonstrated that the N terminal human recombinant (hr) CGA-derived fragments VS-1 (hrCGA1–78) and VS-2 (hrCGA 1–113), as well as the corresponding fragments from bovine CGA and rat CGA (rCGA1-64), exert relevant cardiodepressive and anti-adrenergic influence on several vertebrate (rat, frog and eel) heart preparations (reviewed by Tota et al., 2010). Both VS-1 and VS-2 concentration-dependently inhibit myocardial contractility (negative inotropism) and relaxation (negative lusitropism), thus directly modulating the mechanical performance of intact isolated and perfused hearts beating under basal, i.e., non-stimulated, and adrenergically stimulated, conditions (Tota et al., 2010). Since the VS-1-elicited cardiotropism resulted more relevant than that of VS-2, here we will only focus on VS-1.

The isolated Langendorff-perfused rat heart was used to analyse in detail the VSs influence on myocardial contractility (Cerra et al., 2006). In particular, hrCgA 1–78, containing the VS-1 (CgA 1–76) sequence, at all concentrations tested (11–165 nM) under basal conditions dose-dependently depressed both contractile activity and cardiac work, as indicated by the decrease of left ventricular pressure (LVP) and rate pressure product (RPP: HRx LVP), respectively. No effect on coronary vasomotion was detected, as indicated by the unchanged coronary pressure (CP). Noteworthy, as shown by Pieroni et al. (2007) on the isolated Langendorff rat heart, hrVS-1 is also a negative lusitropic agent since concentration-dependently it decreases myocardial relaxation, i.e., it reduces the maximal rate of the left ventricular pressure decline of LVP [−(LVdP/dt)max], the half time relaxation (HTR), and T/−t ratio obtained by +(LVdP/dt)max/−(LVdP/dt)max. The peptide counteracted the β-adrenergic (Isoproterenol, ISO)-induced positive inotropic and lusitropic effects through a non-competitive mechanism. Taken together, these peptide-induced actions point to VS-1 as a relevant modulator of rat heart mechanical performance under basal and adrenergically stimulated conditions. However, these findings beg the question as to whether or not the peptide-elicited cardiac effects are species-specific. To clarify this issue, using rat cardiac preparations (the isolated Langendorff perfused heart and the papillary ventricular preparation), Cerra et al. (2008) analyzed the cardiac actions of the native (rat) CGA 1-64 (rCGA1-64), i.e., a highly conserved cleavage N-terminal site that reproduces the native rat sequence, corresponding to human N-terminal CGA-derived VS-1 (Metz-Boutigue et al., 1993; Helle et al., 2007). Of note, the concentrations used were the same of its precursor, CGA, in the human serum (i.e., normal levels: 0.5–4 nM, neuroendocrine tumors and last stages of chronic HF: >10 nM) (Helle et al., 2007). The study not only confirmed that, the rCgA1-64 fragment from 33 to 165 nM elicited a significant negative inotropic and lusitropic effects without modifying HR, but also demonstrated that the peptide was a coronary vasodilator, as indicated by a significant reduction of CP. Accordingly, this vasoactivity, for the first time detected on an intact whole coronary bed, further corroborated the “vasostatin” (*ad litteram*) profile of this fragment, as previously reported on segments of bovine coronary resistance arteries, intrathoracic artery and saphenous vein exposed to the hrCgA 1–78 active domain 1–40 (Brekke et al., 2002). The absence of coronary activity of the human recombinant hrCgA1-78, previously evidenced by Cerra et al. (2006), suggests that distinct species-specific sensitivities of the vascular tissues toward VS peptides may account for these different responses. This, however, does not appear to be the case of the myocardium, since the comparison of the contractile effects of rCGA 1-64 and hrCGA1-78 highlights their substantial similarity. This suggests that the rat heart may be a useful model for medically-oriented studies regarding the potential of VS−1as a therapeutic agent. The treatment with rCGA65-76 did not modify cardiac performance at all concentrations tested, except for a small chronotropic effect from 11 to 110 nM, and did not modify the β-adrenergic (ISO)-induced intrinsic activity. An important characteristic of rCGA 1-64 is its ability to counteract ISO (1 μM)- and endothelin 1 (ET-1)-elicited positive contractility, as well as the potent ET-1-induced coronary constriction. Experiments on isolated papillary muscles, i.e., an experimental model in which contractility is analyzed independently from HR and coronary flow, confirmed that the peptide depresses basal and ISO-elicited contractility, without affecting calcium transients on isolated ventricular cells.

The analysis of the percentage of variations of LVP, which provides the EC50 values in the presence of either increasing concentrations of ISO alone or of ISO plus rCGA 1-64 (11, 33, and 65 nM), showed that rCGA1-64 elicits its anti-β-adrenergic action through a functional non-competitive antagonism, confirming the results obtained with the human VS-1 (Cerra et al., 2006).

Furthermore, to have an insight on structure-function relationship, three modified peptides were tested on both rat heart and papillary muscles, showing that the di-sulfide bridge was necessary for the cardiac activity.

In the heart, similarly to the hrVS-1 signal-transduction (Cerra et al., 2006), the rCGA1-64 signals through a Gi/o protein-PI3K-NO-cGMP-PKG-dependent mechanism. In particular, as clarified by the results obtained on the isolated papillary muscle, the action mechanism appears to implicate a calcium-independent/PI3K-dependent NO release by endothelial cells. In fact, rCGA1-64, had no effect on intracellular calcium concentration in isolated ventricular cells, but elicited NO release from cultured bovine aortic endothelial cells (BAE-1) through a calcium-independent mechanism. The eventual involvement of the endocardial endothelium in this mechanism remains to be evaluated. The evidence that the rCGA1-64-induced coronary dilation is also abolished by inhibitors of the NO-cGMP-PKG pathway suggests the likely endothelial release of vasodilator autacoids such as NO.

The control of the NO/NOS system on myocardial contractility and its common tonic depressive influence have been extensively documented. In the ventricular myocardium of the rat NOS-generated NO depresses contractility through sGC-PKG mechanism which decreases L-type Ca^2+^ current (Abi-Gerges et al., 2001) and troponin I phosphorylation (Hove-Madsen et al., 1996). Calcium-independent eNOS activation has been shown to take place after stimulation of endothelium with insulin, insulin-like growth factor-1 (IGF-1) and estrogens (Hartell et al., 2005) through the possible involvement of Akt dependent NOS phosphorylation (Shaul et al., 2002). Conceivably, our experiments on papillary muscles and on BAE-1 cells treated with the PI3K inhibitor wortmannin strongly suggest that the rCGA1-64-elicited NO synthesis depends on PI3K activation. Maniatis et al. (2006) have proposed a calcium-independent mechanism of eNOS activation involving caveolae-mediated endocytosis elicited by the albumin-binding protein gp60 and activation of downstream Src, Akt and PI3K pathways. It has been hypothesized that VSs may interact with caveolar domain (see for references, Tota et al., 2007), and that endothelial cells internalize CGA1-78 (Ferrero et al., 2004). Therefore, it is possible that a similar mechanism may explain VS-1 dependent NOS activation in BAE-1 cells, an issue that needs further research. On the whole, these results strongly support our hypothesis (Tota et al., 2008, 2010) that in the rat heart VS-1, and particularly the homologous rCGA1-64 fragment may act as an autocrine/paracrine modulator of myocardial and coronary performance, functioning as homeostatic stabilizer against heightened SAN tone.

## Cardiac actions of CST

CST was initially identified as the most potent endogenous antagonist of nicotinic-cholinergic receptor (nAChR), exerting antagonistic inhibition of nicotine-evoked CAs secretion in a non-competitive way (Mahata et al., 1997). However, it has revealed itself to act as a multifunctional peptide with different action mechanisms. The *in vitro* and *in vivo* vasoactive and relevant anti-hypertensive properties of CST have been extensively reviewed (e.g., Mahata et al., 2010) and will not be considered here.

**Figure 3:**
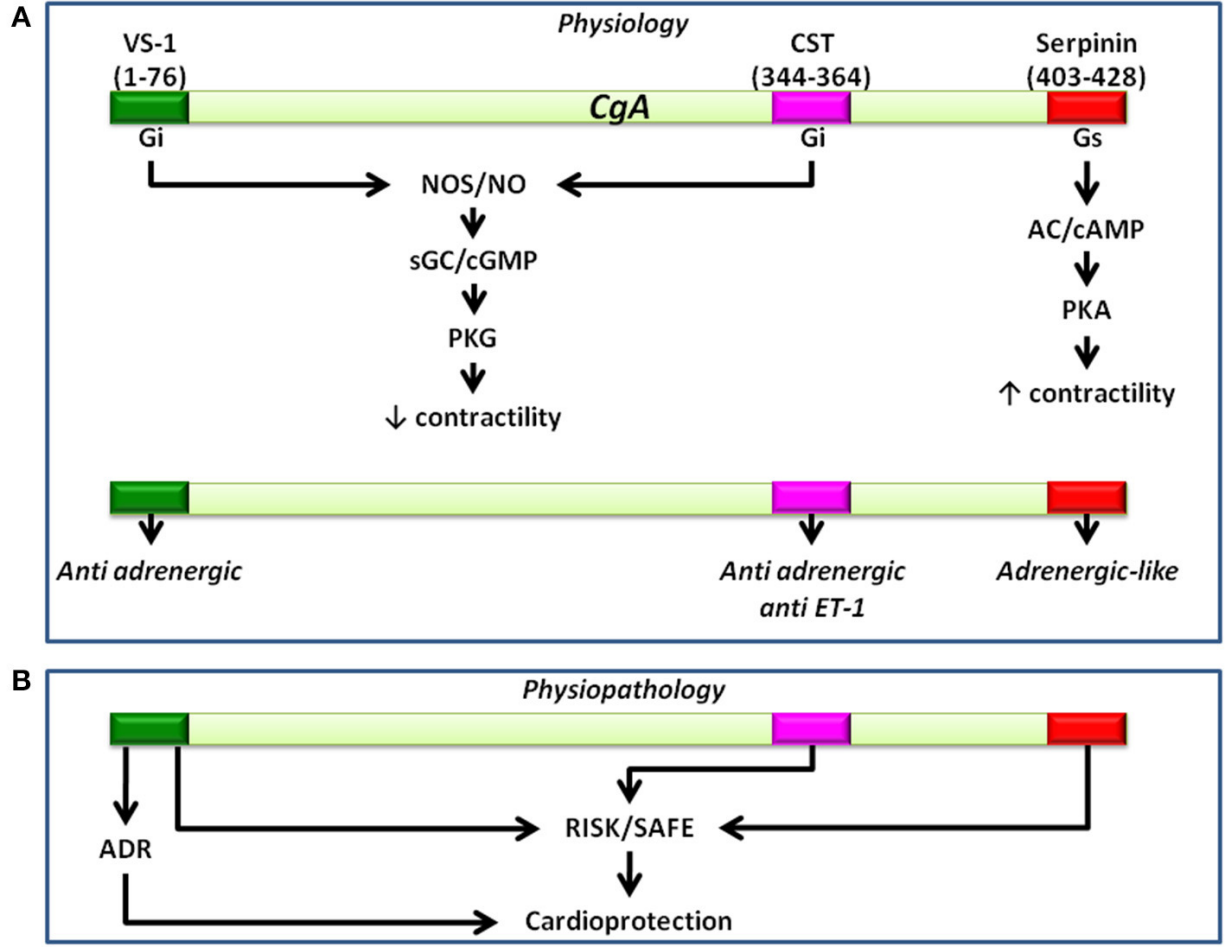
**Representative scheme showing the physiological (A) and the physiopathological (B) (i.e., cardioprotection) pathways activated by CgA-derived peptides**. Cardioinhibitory effects induced by VS1 and CST involve the NOS/NO/cGMP/PKG pathway. Serpinin-dependent positive inotropism involves the AD/cAMP/PKA pathway.

The cardiotropic actions of wild-type CST (WT-CST, human CGA352-372) and its naturally occurring variants (G364S-CST and P370L-CST) were demonstrated for the first time by Angelone et al. (2008) who used the isolated and Langendorff perfused rat heart to evaluate the peptide-induced cardiac effects independently from the minute-to-minute control exerted by SAN over cardiac output (CO) and vascular tone. WT-CST (from 11 to 200 nM) dose-dependently decreased left ventricular pressure (LVP, index of contractility), rate pressure product (index of cardiac work) and both positive and negative LVdP/dt (index of maximal rate of left ventricular contraction and relaxation, respectively), while increased CP. While G364S-CST was ineffective on basal mechanical performance, P370L-CST elicited only a negative inotropism. Human CST variants counteracted the positive β-adrenergic (ISO)-induced inotropic and lusitropic effects with different rank order of potency (for the ISO-induced positive inotropism: WT-CST>G364S-CST>P370L-CST; for the ISO-induced positive lusitropism: G364S-CST>WT-CST>P370L-CST (Angelone et al., 2008). In both the isolated Langendorff rat heart (Angelone et al., 2012b) and in rat papillary muscle (Bassino et al., 2011), CST before eliciting these major prolonged myocardial actions, induced an early transient positive inotropic effect that disappeared after 5 min from administration. This effect was mediated by H1 histamine receptors, since it was abolished by H1 blockade, consistent with previous evidence that in the rat the activation of myocardial H1 receptors mediates the histamine-dependent positive inotropism (Matsuda et al., 2004). The relevant tonic CST-induced negative inotropism and lusitropism are attained through pertussis toxin-sensitive (PTX), receptor-independent activation *via* heterotrimeric G proteins and Gαi/o subunits. Likewise, Gi/o proteins activation could limit the Gs-mediated positive contractile effects. The peptide signaling involves both beta2-AR and beta3-AR, with a higher affinity for the first one (as evidenced by low IC50 values) but not beta1-AR, being unaffected by cholinergic receptor inhibition, respectively (Angelone et al., 2008). It is known that beta1-AR, coupled to Gs proteins, is responsible for positive inotropism and lusitropism, while beta2-AR, mainly coupled to Gi/o proteins, is responsible for the opposite effects on contractility and relaxation (Xiao et al., 1999). Moreover, cardiac beta2-AR/Gi stimulation can activate PI3K with consequent negative inotropism (Yano et al., 2007). Interestingly, an important component of the CST signal-transduction is represented by the PI3K/Akt/eNOS/NO/cGMP-dependent pathway, as shown by the PI3K blockade which abolishes the peptide-induced inotropism and lusitropism (Angelone et al., 2012b). The CST signaling also requires an endothelium-derived bioactive NO mechanism, since it depends from the functional integrity of the endothelium. To further analyse the signal-transduction mechanism of CST and its variants, Bassino et al. (2011) measured contractility and Ca^2+^ transients respectively on papillary muscles and isolated cardiomyocytes in basal conditions and after beta-adrenergic stimulation, evaluating on BAE-1 NO production and eNOS phosphorylation (P^Ser1179^eNOS). Their data show that CST dose-dependently (5–50 nM) reduces the effect of beta-adrenergic stimulation which, rather than resulting from a direct myocardial action of the peptide, depends from a Ca^2+^-independent/PI3K-dependent NO release from endocardial endothelial cells. Consistent with this, CST induces in BAE-1 cells a Wortmannin-sensitive, Ca^2+^-independent increase of NO production and P^Ser1179^eNOS. The variant P370L-CST, but not G364S-CST, exerted an anti-adrenergic effect and an increased NO release comparable to that elicited by WT-CST. As mentioned above (*Cardiac actions of VS-1*), such calcium-independent, caveolae-mediated endocytosis mechanism for activation of Akt-PI3K-eNOS pathway, also proposed for insulin, insulin-like growth factor-1, and estrogens, has been suggested for VS-1 (Ramella et al., 2010 and references therein). In agreement with the NO-sGC-cGMP-dependent signaling, in the peptide-treated hearts cGMP was significantly increased and at least two of its targets appeared implicated in the CST action mechanism. One is PKG, known to depress myocardial contractility by reducing both L-type Ca^2+^ current and troponin C affinity for calcium (Angelone et al., 2012b). The other target is phosphodiesterases type 2 (PDE2) (Angelone et al., 2012b). Its selective inhibition by EHNA abrogates the CST-induced inotropic and lusitropic effects both under basal and stimulated (ISO) conditions, indicating a relevant PDE2 involvement. Conceptually important in the context of temporal SAN/CGA interactions, Angelone et al. (2012a) also demonstrated the involvement of both phospholamban (PLN) and beta-arrestin S-nitrosylation. beta-arrestin is implicated in the desensitization and internalization of G-protein-coupled receptors (including beta1-AR), while its S-nitrosylation elicits and accelerates beta1-AR desensitization (Ozawa et al., 2008). PLN controls sarcoplasmic reticulum Ca-ATPase (SERCA2a) by a phosphorylation/dephosphorylation mechanism so that while dephosphorylated PLN inhibits SERCA2a-dependent SR Ca^2+^ sequestration (Reddy et al., 1999), phosphorylated PLN at Ser16 by PKA relieves its inhibition on SERCA2a (Schmidt et al., 2001). Thus, PLN S-nitrosylation appears a selective target of the CST-induced NO release able to regulate SR Ca^2+^ fluxes and Ca^2+^ availability for inotropy and lusitropy. These data highlight a temporally tuned and more sophisticated CST-promoted anti-beta-adrenergic cardiac modulation than previously perceived, which consists in rapid (PDE2 and PLN) and medium-term (beta-arrestin-mediated beta1-AR desensitization) regulatory switches. Accordingly, a PDE2-dependent short-term signal switches off β adrenergic activity, while a medium-term signal promotes more prolonged adrenergic counteraction through beta-arrestin-mediated beta1-AR desensitization. In the rat heart the coronary profile of CST appears non-univocal. CST under basal conditions dose-dependently increases CP (maximum response at 200 nM) and abolishes the ISO-dependent vasodilation; in contrast, it potently vasodilates the ET-1 preconstricted coronaries (Angelone et al., 2008), confirming the vasodilation promoted by exogenous CST in human subjects (O'Connor et al., 2002; Fung et al., 2010). Since it is known that in mammals the stimulation of cardiac ETB subtype receptors promote an endothelium-dependent negative inotropism (Brás-Silva and Leite-Moreira, 2005), CST signal was tested in presence of the selective ETB antagonist BQ788. The consequent abrogation of the ET-1- induced effects demonstrated the relevant ET-1/CST cross talk, suggesting at the same time that the CST signal at plasma membrane level significantly converges on the sympatho-inhibitory NO pathway. In conclusion, this growing evidence indicates that CST, in addition to its antihypertensive profile, directly modulates myocardial and coronary functions under both basal and stimulated (β-adrenergic and ET-1) conditions. The peptide-induced negative contractility/relaxation influence, as well as its coronary vasomotion might be viewed as relevant components of a homeostatic counteraction against heightened SAN over-activation, e.g., prolonged stress, HF, hypertensive cardiomyopathy, namely, conditions characterized by a potentially harmful spill-over of CAs, ET-1 and RAS agonists. Conceivably, these cardiac properties of the peptide, together with its antihypertensive and vasoactive profile, suggest that CST can function as a novel autocrine-paracrine modulator cooperating with full-length CGA and its derived VS-1 in the multilevel processes required for cardio-circulatory homeostasis (see Figure 3A).

## Cardioprotective influence of VS-1 and CST

Reperfusion is indispensable for salvaging viable myocardium, following an acute heart infarction. Both ischemic preconditioning (PreC) and post-conditioning (PostC) have become standard procedures for evaluating the cardioprotective ability of an agent when applied respectively before or after an infarcting ischemia can enhance heart function recovery, limiting infarct size (Hausenloy, 2009, 2012; Penna et al., 2009). Various peptides can elicit cardioprotection, triggering both pharmacological pre- and post-conditioning signaling pathways. In rodents, these include the *Reperfusion Injury Salvage Kinase* (RISK) and the *Survivor Activating Factor Enhancement* (SAFE) pathways (Sivaraman et al., 2007; Hausenloy et al., 2011). The actions of Survival kinases, such as phosphatidylinositol-3-kinase (PI3K)/protein kinase B (Akt/PKB) and protein kinase C (PKC), may converge on downstream mitochondrial targets to open ATP-sensitive potassium (mitoKATP) channels, thereby affecting cellular survival through a decrease of necrosis and apoptosis (Zatta et al., 2006; Boengler, 2011). Cardioprotection involves endothelial and endothelial derived NO, as well as adrenergic components (Bell and Yellon, 2003; Pagliaro et al., 2003; Cappello et al., 2007). The acknowledged anti-adrenergic and endothelial PI3K-induced NO signaling of both VS-1 and CST have addressed several studies to verify their eventual cardioprotective profiles. The comparison of the cardioprotective effects of VS-1 and CST in ischemic conditioning highlights a remarkable similarity and subtle differences. VS-1 appears to act as a pre-conditioning inducer while CST acts as a post-conditioning agent (reviewed by Penna et al., 2012). Human recombinant VS-1 protects against the extension of myocardial infarction converging on PKC through two different pathways, one mediated by adenosine A1 receptors and the other mediated by NO release (Cappello et al., 2007). On the other hand, the CST-induced protection reduces infarct size and improves post-ischemic cardiac function *via* PI3K/Akt, PKCs and mito-KATP channel activation, which may implicate a ROS signaling (Perrelli et al., 2013). CST is also able to exert direct myocardial protection *via* an endothelium-independent mechanism, as shown by Penna et al. (2010) in isolated adult cardiomyocytes exposed to simulated I/R, where the peptide has been shown to induce a partial mitochondrial depolarization (Perrelli et al., 2013) (see Figure 3B). Very recently, it has been also reported that CST protects in the post-ischemic SHR heart by increasing the expression of anti-apoptotic and pro-angiogenic factors, supporting its potential therapeutic role, even in the presence of comorbidities, such as hypertension and cardiac hypertrophy (Penna et al., 2014a, June Accepted). These data are of interest also taking into account the angiogenic properties of CST reported by Theurl et al. (2010). A CST-induced S-nitrosylation of calcium channels in the post-ischemic phase has been recently reported by Penna et al. (2014a). Of note, this posttranslational modification of a L-type calcium channel subunit has already been described in preconditioning cardioprotection by Murphy et al. (2014) and in non-ischemic hearts treated with CST by Angelone et al. (2012b). The CST-Post-elicited S-nitrosylation of calcium channel may be functionally important, since the oxidative/nitrosative signaling is known to play a major role in cardioprotection against ischemia/reperfusion injury in both preconditioning and post-conditioning (Pagliaro et al., 2011; Tullio et al., 2013; Penna et al., 2014b).

## Cardiac actions of serpinin

The recent story of serpinin began with Kim and Loh (2006) examining the processing of the C-terminal domain of CGA in mouse pituitary cell line, (AtT-20). They showed that a CGA fragment, but not intact CGA, secreted in an activity-dependent manner, increased granule biogenesis by up-regulating protease nexin-1 (PN-1), a serine protease inhibitor protein, which then stabilizes granule proteins to enhance their levels in the Golgi complex. Successively, Koshimizu et al. (2010, 2011) demonstrated that, in AtT-20 pituitary cells, both mouse CGA435–460, a synthetic 26 amino acid residue peptide named serpinin, and endogenous-serpinin related peptides were able to induce PN-1 mRNA up-regulation. In particular, they identified in AtT-20 cell-conditioned medium a 23-mer serpinin-like fragment, pyroglutaminated (pGlu-23Leu) serpinin, which was present in the highest amount compared to the other serpinin-related peptides serpinin (Ala26Leu), and serpinin-Arg-Arg-Gly (Ala29Gly), (Koshimizu et al., 2011). The recognition that the serpinin region of CGA is highly conserved in mammals suggests that peptides derived from this domain may have relevant physiological functions.

pGlu-serpinin upregulates PN-1 mRNA expression in AtT-20 cells *via* a cAMP-protein kinase and exerts an antiapoptotic effect on these cells and on cultured CNS neurons exposed to oxidative stress. Since PN-1 expression is upregulated by CGA in AtT-20 cells (Kim and Loh, 2006) and PN-1 is a potent inhibitor of plasmin which causes apoptosis in chronically injured neurons (Ho-Tin-Noé et al., 2009), it is possible that pGlu-serpinin can be implicated in plasmin inhibition.

Soon after, Tota et al. (2012) demonstrated the involvement of serpinin in heart function. Using HPLC and ELISA methods, they detected in the rat heart serpinin peptides, Ala29Gly and pGlu-serpinin being the predominant fragments. This means that the rodent heart is able to process the C terminal domain of CGA as it does with its N terminal domain. Moreover, using the Langendorff perfused rat heart to evaluate the hemodynamic responses, Tota et al. (2012) showed that serpinin and pGlu-serpinin dose-dependently (11–165 nM) increase inotropism and lusitropism within the first 5 min after administration. pGlu-serpinin appears more potent than serpinin, its action starting from 1 nM. These effects were corroborated by the results obtained on the isolated rat papillary muscle preparation which allows to measure contractility in terms of tension development and muscle length. It was found that while pGlu-serpinin induces positive inotropism, Ala29Gly is unaffective. Both pGlu-serpinin and serpinin act through a beta1-Adrenergic Receptor/Adenylate Cyclase/cAMP/PKA pathway (Tota et al., 2012), a finding of particular interest in view of the opposite beta-depressive profile of the two CGA-derived peptides, VS-1 and CST. The beta-adrenergic-like agonist profile of serpinin is further confirmed by the results showing that pGlu-serpinin increases intracardiac cAMP levels. pGlu-serpinin and serpinin at nanomolar range act as beta 1-adrenergic-like agonists, mimicking the intracardiac sympathetic neurotransmitters and/or circulating CAs. Like these agents, they increase cellular levels of cAMP, thereby remarkably affecting myocardial mechanical performance. Namely, they increase the rate and extent of tension development during systole (positive inotropy), hence augmenting stroke volume; at the same time, they accelerate myocardial relaxation (positive lusitropy), hence shortening the overall duration of diastole. As demonstrated by the experiments on isolated papillary muscle, the serpinin action is independent from any possible alteration in HR and coronary flow rate, as well as from norepinephrine release from sympathetic nerve terminals (as demonstrated by tyramine treatment). Studies are needed to clarify the earliest events underpinning the transduction of the serpinin signal. Since no receptor or direct binding partner has been so far identified for serpinin or pGlu-serpinin, on the basis of the reported functional antagonism, Tota et al. (2012) have proposed that serpinin and pGlu-serpinin may function as allosteric modulators of the beta-adrenergic receptor independently from the ligand binding site, thereby triggering the well-known beta-adrenergic-induced cascade. Therefore, serpinin peptides resemble the other CGA-derived peptides in such apparent lack of classical receptors. According to a mechanism common for cAMP elevating agonists (Koshimizu et al., 2010), serpinin and pGlu-serpinin might bind to a G protein-coupled receptor (GPCR) enhancing the cardiac cAMP levels, with consequent hemodynamic effects that are indeed abolished by selective inhibition of AD and PKA. The major downstream targets of the AD-cAMP signaling are PKAs which phosphorylate a number of proteins, including SERCA and its associated modulatory protein, PLN, both crucial regulators of myocardial inotropy and lusitropy (Katz, 1990). SERCA-dependent Ca^++^ uptake within SR promotes cation removal during diastole, thus affecting relaxation and the subsequent contraction (Satoh et al., 2011). Noteworthy, blockade of SERCA activity by thapsigargin, abolishes the pGlu-serpinin-elicited inotropy and lusitropy. It is well known that Ca^++^ sensitivity for SERCA is enhanced by PKA-induced phosphorylation of PLN, a downstream target of the beta-adrenergic-PKA cascade which plays a determinant role in the adreno-sympathetic modulation of myocardial contractility/relaxation (Mattiazzi et al., 2007). It is thus physiologically relevant that pGlu-serpinin induces PNL phosphorylation at Ser16, residue (see Figure 3A).

### Putative cardioprotective influence

The pGlu-serpinin-PKA cascade also appears to induce phosphorylation of ERK1/2 and GSK3beta known to mediate PGE2 and EP4 signaling in neonatal ventricular myocytes (Tota et al., 2012 and references therein). These proteins are components of the protective RISK (*reperfusion injury signaling kinase*) pathway implicated in myocardial protection against ischemia-reperfusion injury in rodents (Hausenloy et al., 2011) and, possibly, in the pGlu-serpinin-induced anti-apoptotic effects against reactive oxygen species (ROS) reported in cultured cerebral neurons by Koshimizu et al. (2011). This observation has prompted preliminary experiments aimed to test the cardioprotective influence of serpinin against I/R injury in the rodent heart. Very recently, Tota et al. (2012) reported that, given in pre- and post-conditioning, pGlu-serpinin reduces infarct size and preserves the hemodynamic function of both isolated normotensive and SHR hearts, being more protective in the latter. Moreover, in both normotensive and hypertensive hearts, pGlu-serpinin while inducing a mild cardioprotection in pre-conditioning, exerts streaking cardioprotection in post-conditioning. All these effects appear to involve the protective RISK pathway (Tota et al., 2012) (see Figure 3B).

**Figure 4:**
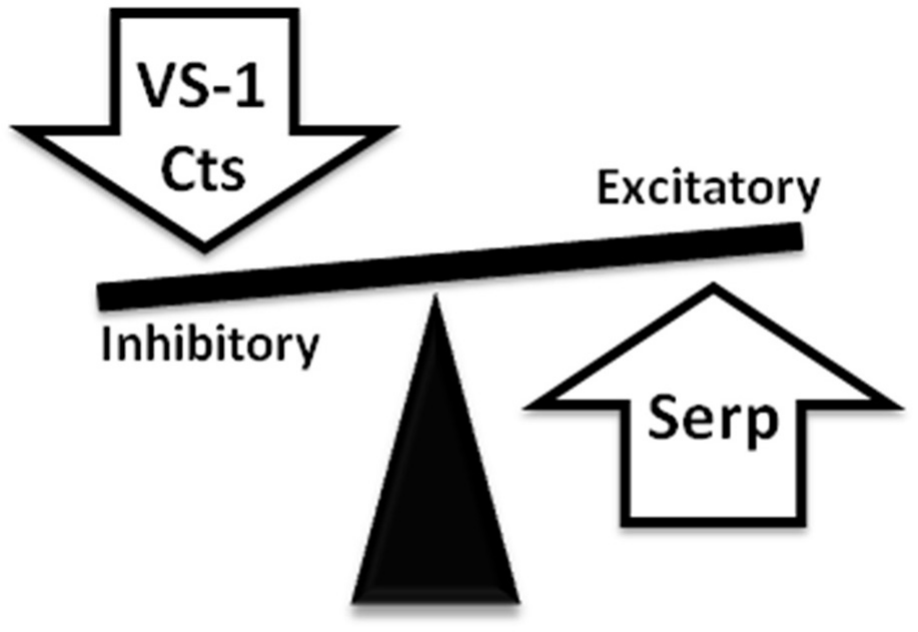
**Representative picture showing the ability of CgA-derived peptides to reset cardio-circulatory homeostasis through “whip-brake” networks in response to perturbed conditions**. A tissue-specific and spatio-temporally concerted processing of CGA can generate counter-regulatory peptides able to reset cardio-circulatory homeostasis through “whip-brake” networks.

In conclusion, the recent recognition that the C terminal domain of CGA can generate in the heart serpinin fragments that enhance myocardial contractility/relaxation through beta1-adrenergic/AD/cAMP signaling adds new evidence to the sympatho-chromaffin profile of CGA. It is likely that in response to perturbed conditions, a tissue-specific and spatio-temporally concerted processing of CGA can produce counter-regulatory peptides able to reset cardio-circulatory homeostasis through “whip-brake” networks (see Figure 4). It might be expected that such ability of CGA and its derived VS-1, CST and serpinin could be differentially regulated in relation to short- or long-term activation of SAN, but this issue is still a closed book. In this context, the evidence that pGlu-serpinin acts on beta1-AR in a functional manner, i.e., independent of a direct classical interaction with AR active site, may be of putative therapeutical interest, being an alternative to direct adreno-receptor stimulation when this can be undesirable. In fact, it is known that prolonged, long-term beta-AR stimulation can directly induce maladaptive cardiac hypertrophy, eventually, leading to HF.

## Conclusions and perspectives

Studies in the past 15 years have convincingly revealed the cardio-vascular activities of CGA and its derived peptides VS-1, CST and serpinin, along with their striking adreno-sympathetic regulatory influences (see Table 1). This knowledge, which has widened the prohormone/cytokine profile of CGA, has been paralleled and integrated by a growing number of clinical studies that have documented the biomedical implications of CGA and its peptides (especially the antihypertensive CST) in various cardiovascular diseases, particularly in relation to their diagnostic and prognostic value. It is well known that conditions characterized by perturbed cardio-circulatory homeostasis, especially in the case of severe heart diseases (i.e., myocardial infarction, acute coronary syndromes, ventricular hypertrophy), activate complex neuro-humoral networks (SAN and NPs, to mention two), finely integrated at both local and systemic levels, that tend to re-establish the constancy of the internal milieu (Angelone et al., 2012a and references therein). As illustrated in this review, the available evidence strongly supports the view that CGA and its cardiotropic fragments represent an important component of these neuroendocrine networks, challenging cross-disciplinary contributions in this direction. However, the excitement that accompanies this new knowledge is paired with a deeper perception of the many questions that need to be clarified. We neither know to which extent the extensive *ex vivo* evidence can be extrapolated to the *in vivo* situation, nor if the peptide-induced cardiac actions are beneficial for the diseased heart or they may concur to aggravate its pathology. For example, in acute coronary syndromes the vasodilatory, negative inotropic and lusitropic properties, and the anti-adrenergic effect elicited by the two CGA fragment VS-1 and CST, might be either detrimental, thus contributing to HF development, or compensatory, at least at the beginning of the pathology (Kubota, 1997). Similarly, in the context of circulatory homeostasis, there is a need to deepen the potential beneficial role of the CGA/NO axis in relation to the CGA-induced “anti-inflammatory” ability and protection of the endothelial barrier against TNF-alpha-induced vascular permeability (Ferrero et al., 2002). At the same time, an open window remains concerning the putative value of CGA and its fragments as cardiovascular diagnostic/prognostic biomarkers. In this regard, caution is especially needed because of two main reasons. The first is to understand whether these peptides provide incremental information with respect to conventional biomarkers. The second is to prevent errors in evaluating CGA and its fragments in biological samples by developing appropriate and sensitive methods for their detection and measurements, excluding interference induced by food intake and drug therapies (i.e., proton pump inhibitors, H2 antagonists, and glucocorticoids (Giusti et al., 2004).

**Table 1:** **Synopsis of the cardiac effects of CgA and its derived peptides**.

**Peptide**	**Tissue**	**Contractility/Relaxation**	**Heart rate**	**Vasoactivity**	**Doses**	**Adrenergic Stimulation**	**References**
CgA	*Ex vivo rat* heart	Reduction	No changes	Vasodilation	1 pM ÷ 4 nM	-	Pasqua et al., 2013
VS1	*Ex vivo rat* heart	Reduction	No changes	No changes	11 ÷ 165nM	Non-competitive	Cerra et al., 2008
						Antagonism	
rCGA1-64	*Ex vivo rat* heart	Reduction	No changes	Vasodilation	33 ÷ 165 nM	Non-competitive	Cerra et al., 2008
	Rat Papillary muscles				10 ÷ 100 nM	Antagonism	
CST	*Ex vivo rat* heart	Reduction	No changes	Vasodilation	11 ÷ 200 nM	Non-competitive	Angelone et al., 2008, 2012a
						Antagonism	
Serpinin	*Ex vivo rat* heart	Increase	No changes	No changes	11 ÷ 165 nM	Beta-adrenergic like inotropim	Tota et al., 2012
	Rat Papillary muscles				1 ÷ 33 nM		

The following years will tell us how can our existing view of CGA biology be modified in face of the new discoveries and how are we to deal with the cross-disciplinary challenges provided by the upcoming research.

### Conflict of interest statement

The authors declare that the research was conducted in the absence of any commercial or financial relationships that could be construed as a potential conflict of interest.
